# The Impact of Parental Role Distributions, Work Participation, and Stress Factors on Family Health-Related Outcomes: Study Protocol of the Prospective Multi-Method Cohort “Dresden Study on Parenting, Work, and Mental Health” (DREAM)

**DOI:** 10.3389/fpsyg.2019.01273

**Published:** 2019-06-12

**Authors:** Victoria Kress, Susann Steudte-Schmiedgen, Marie Kopp, Anke Förster, Caroline Altus, Caroline Schier, Pauline Wimberger, Clemens Kirschbaum, Tilmann von Soest, Kerstin Weidner, Juliane Junge-Hoffmeister, Susan Garthus-Niegel

**Affiliations:** ^1^Department of Psychotherapy and Psychosomatic Medicine, Faculty of Medicine of the Technische Universität Dresden, Dresden, Germany; ^2^Institute of Biological Psychology, Faculty of Psychology of the Technische Universität Dresden, Dresden, Germany; ^3^Department of Gynecology and Obstetrics, Faculty of Medicine of the Technische Universität Dresden, Dresden, Germany; ^4^Department of Psychology, PROMENTA Research Center, University of Oslo, Oslo, Norway; ^5^Department of Child Health, Norwegian Institute of Public Health, Oslo, Norway

**Keywords:** parental mental health, work participation, role distribution, peripartum stress, DREAM study, hair cortisol, multi-method approach, study protocol

## Abstract

The Dresden Study on Parenting, Work, and Mental Health (“**DR**esdner Studie zu **E**lternschaft, **A**rbeit, und **M**entaler Gesundheit”, **DREAM**) aims to prospectively investigate the relationship between parental work participation, role distribution, stress factors, and their effects on perinatal outcomes and long-term family mental and somatic health in a community sample targeting *N* = 4,000 individuals, i.e., 2,000 couples, expecting a child and residing in Dresden, Germany (interim sample of *N* = 1,410 participants, recruitment ongoing). Various questionnaires are completed at four measurement points from pregnancy to 2 years postpartum (prolongation into middle childhood planned). Applying a multi-method approach, long-term endocrinological data (analyses of hair cortisol concentrations and other endogenous hormones, “DREAM_HAIR_”) and qualitative interview data (regarding gender role attitudes and distribution of domestic work, child care, and paid employment; “DREAM_TALK_”) are obtained. In this study protocol, the theoretical background, methods, and preliminary results considering sociodemographic characteristics during pregnancy and birth-related factors at 8 weeks postpartum are presented. Additionally, there is a focus on our endocrinological sub-study DREAM_HAIR_. In this sub-study currently comprising *N* = 152 participants, i.e., 88 families (recruitment ongoing), we want to gain knowledge on the transgenerational processes of stress regulation and psychopathology in the whole family by analyzing hair cortisol concentrations in both parents and children during the course from pregnancy (or after birth regarding children) to at least 2 years postpartum. By comparing data of the community sample to a clinical sample of mothers with postpartum mental disorders, their children, and their partners during the period between admission and discharge from a mother-baby unit and post-treatment (“DREAM_MBU_”), the course of mothers' psychopathology, parent-infant interaction, and infant regulation disorders with special regard to long-term endocrine correlates will be examined. With previous studies neglecting the fathers or partners involved, a major advantage of DREAM is the use of a multi-method and multi-level approach by examining the whole family in a longitudinal design. Therefore, the DREAM study will contribute to a better understanding of the role of social, work, and stress factors for mental and somatic health and its long-term endocrine correlates in the natural course of becoming a family.

## Introduction

Expecting a child marks a transition involving several physiological, psychological, and structural changes for the individual as well as for the couple. The majority of German women reduces its work participation after giving birth to a child and continues staying at home after paid parental leave runs out 12 months postpartum (or after the stretched out parental leave ends 2 years postpartum for families who worked part-time for a while); and of those women who do return to their jobs, the majority only works part-time (Bundesministerium für Familie Senioren Frauen und Jugend, 2017a). German mothers are eligible to split the parental leave with the father and if the fathers take at least two so-called “fathers' months” of paid leave, the parental allowance is extended to 14 months in total. Although the amount of fathers taking paternal leave is increasing (with roughly one third taking the “fathers' months”), men usually stay at home for a shorter period compared to women and continue working the same amount of time afterwards (Bundesministerium für Familie Senioren Frauen und Jugend, 2017a). This imbalance is aggravated by several cultural reasons (e.g., when it is appropriate to work again and concerning distribution of labor between sexes) and structural reasons like the tax system (i.e., the German tax system penalizes married couples economically when both partners share domestic work, child care, and paid employment equally, leading to a polarization of working time), a cash-for-care benefit (“Betreuungsgeld”; which subsidies parents (mostly women) staying at home with their children until they turn 3 years old), and an insufficient access to day care. Short- and long-term consequences of mothers' longer parental leave and not being employed refer to an unbalanced distribution of domestic work and child care (Buehler and O'Brien, 2011; Schober and Zoch, 2015), unequal wages after re-entry in the labor market (Davies and Pierre, 2005; Bryan and Sevilla-Sanz, 2010), and a delay in their professional career which may result in an increasing dependency on the partner and consolidate a gender gap interfering with gender equality (Barker and Pawlak, 2011; Miani and Hoorens, 2014).

Beyond equality considerations, the impact of paid employment on health has to be considered. In this context two contrary hypotheses have been suggested: the role strain or scarcity hypothesis postulates that additional roles like being a working woman impair maternal health due to additional daily hassles and demands (Goode, 1960). Thus, it may be more challenging to care for oneself and for a child and meet its needs. In contrast, the role enhancement hypothesis suggests that women with several roles are healthier because of positive stimulating input in their professional life and better access to resources that help in dealing with these demands (Sieber, 1974; Marks, 1977). As a consequence, the mother may have more energy to dedicate herself to family life in her spare time. In accordance with the latter, there is recent evidence that maternal work participation may have a positive influence on mental and somatic health (Klumb and Lampert, 2004; Buehler and O'Brien, 2011; Frech and Damaske, 2012; Cruise et al., 2018). Nevertheless, further results suggest a negative (in line with the scarcity hypothesis) or missing association between maternal work participation and the mother's mental and somatic health (Schwab-Reese et al., 2017; Liu et al., 2018). Consistent with findings on negative associations, women have been found to have a greater risk for suffering from work-privacy conflict than men probably due to multiple private and occupational burdens (Garthus-Niegel et al., 2016). Thus, it may be hard to put compatibility of work and family into practice. In sum, the potential etiologic role of employment on health warrants further investigation. Indeed, as the above mentioned evidence mainly comes from cross-sectional studies conducted in the United States, it remains unclear whether the previous research results are applicable to working women and families in Europe or even Germany, where maternal work participation is comparatively low. Moreover, previous evidence also neglected the role of important confounding or moderating factors, e.g., precarious working conditions and psychosocial work stress (Klumb and Lampert, 2004; Buehler and O'Brien, 2011), despite of recent evidence indicating their negative impact on parents' mental and somatic health (Caparros-Gonzalez et al., 2017; Philpott et al., 2017).

Chronic effects of stress on mental and somatic health, e.g., caused by precarious working conditions or psychosocial work stress, have been closely linked to the activity of the body's stress response systems, particularly the hypothalamic-pituitary-adrenal (HPA) axis leading to the secretion of cortisol (Chrousos, 2009). Studies investigating the relationship between work participation or precarious working conditions and cortisol are predominantly based on traditional cortisol measures. For example, unemployment has been found to be linked with alteration of diurnal or overall cortisol secretion, e.g., in blood (e.g., Arnetz et al., 1991; Maier et al., 2006) or saliva (e.g., Grossi et al., 2001; Gallagher et al., 2016). The direction of findings is characterized by some inconsistency (review: Sumner and Gallagher, 2017), e.g., with some studies indicating elevated (e.g., Arnetz et al., 1991) or lowered (e.g., Gallagher et al., 2016) overall cortisol levels in unemployed people compared to employed people, while some studies failed to show such a difference (e.g., Ockenfels et al., 1995). Regarding the relations between psychosocial work stress on the activity of the HPA axis, study results support the relevance of endocrine correlates as a mediator of the impact of work stress on health although findings are characterized by a notable heterogeneity (review: Siegrist and Li, 2017). Part of the reason for mixed results may be due to limitations in the assessment of long-term cortisol secretion. Specifically, previous cortisol assessment methods particularly reflect short-term secretory activity over periods ranging from minutes (saliva, plasma) to hours (urine; Stalder et al., 2017). Given that acute cortisol secretion is highly volatile and affected by a range of situational factors (Stalder and Kirschbaum, 2012), these methods provide rather unreliable estimates of long-term cortisol output. The analysis of hair cortisol concentrations (HCC) constitutes a relatively recent tool that may increase the quality of the assessments of long-term cumulative cortisol levels in such research. Through an incorporation of lipophilic substances into the slowly growing hair matrix, HCC are supposed to be a non-invasive and easily obtainable retrospective marker of cortisol levels integrated over the previous months (reviews: Stalder and Kirschbaum, 2012; Stalder et al., 2017). Further advantages of hair cortisol analysis include the robustness to acute situational influences and the independency from non-compliance issues (Russell et al., 2012; Stalder and Kirschbaum, 2012; Stalder et al., 2017). Over the past years, considerable evidence has emerged in support of the general validity and reliability of hair cortisol analysis (review: Stalder and Kirschbaum, 2012). Specifically, recent studies have shown positive associations between HCC and cumulative cortisol data from repeated assessments using traditional cortisol measures (Sauvé et al., 2007; D'Anna-Hernandez et al., 2011; van Holland et al., 2012; Short et al., 2016). Further indirect support stems from research that found a correspondence of HCC data and the expected secretory pattern in conditions with well-known endocrine alterations (review: Stalder et al., 2017). For example, the well-known pattern of increasing cortisol levels over the course of pregnancy was confirmed by HCC data (Kirschbaum et al., 2009; D'Anna-Hernandez et al., 2011; Karlén et al., 2013; Hoffman et al., 2016).

However, evidence for HCC in relation to work participation and precarious working conditions remains rare and inconsistent. Some studies indicate links between HCC and unemployment (Dettenborn et al., 2010), job insecurity (Herr et al., 2017), and shift work (Manenschijn et al., 2011). Other studies have come to opposite findings underlining the need of further investigations on larger samples taking into account moderating and mediating factors within a longitudinal design (Janssens et al., 2017; van der Meij et al., 2018). Furthermore, HCC were found to serve as a marker of stress in several contexts, e.g., stress-related somatic conditions (Pereg et al., 2011; Manenschijn et al., 2013; Stalder et al., 2013; Kuehl et al., 2015) and mental disorders (review: Staufenbiel et al., 2013; Wester and van Rossum, 2015; Stalder et al., 2017; Steudte-Schmiedgen et al., 2017). Evidence suggests that HCC sensitively reflect clinical and/or stress-related conditions. For example, higher HCC were detected in patients with major depression (Dettenborn et al., 2012; Hinkelmann et al., 2013; Wei et al., 2015) and late-onset bipolar disorder (Manenschijn et al., 2012) while attenuation was seen in generalized anxiety disorder (Steudte et al., 2011) or posttraumatic stress disorder characterized by a long-term time-interval since traumatization (Steudte et al., 2013). Still, meta-analytic data revealed no consistent relationships with questionnaire-based measures of perceived stress or clinical symptoms (Stalder et al., 2017). This seems also to be evident among pregnant women, i.e., HCC were not consistently found to be related to prenatal psychological distress (review: Mustonen et al., 2018). Interestingly, Bowers et al. (2018) found an association between self-reports of distress and HCC during pregnancy only in women who experienced high levels of childhood adversity compared to women without such experiences. This supports the notion that early adversity contributes to the long-term activity of the HPA axis. Here, a longitudinal investigation of HCC over an extended period of time is warranted. However, the fact that HCC were more frequently related to the number of self-reported stressful or negative life events (Karlén et al., 2011, 2015; Grassi-Oliveira et al., 2012; Staufenbiel et al., 2014; Steudte-Schmiedgen et al., 2015) or traumatic events across the lifespan (Steudte et al., 2013; Steudte-Schmiedgen et al., 2016) suggests that stronger psychoendocrine correspondence may be achieved by using stress measures based on objectives criteria.

So far, only implications for the mother's health herself have been considered in this paper. More importantly, stress factors, e.g., precarious working conditions and psychosocial work stress, may not only be essential for the health of the mother herself, but also play a major role for the entire family and especially for development and health of the offspring (Lucas-Thompson et al., 2010; Jaursch and Lösel, 2011). In fact, the early environment is fundamental for long-term mental and somatic health of a child (Van den Bergh et al., 2017). Child outcomes have been found to be negatively affected by maternal mental disorders (Pearson et al., 2013; Gentile, 2017) and self-reported stress (Wadhwa et al., 2011; Van den Bergh et al., 2017) during pregnancy.

Long-term effects of maternal adversity on the offspring are referred to as fetal programming with a transgenerational transmission of alterations of the long-term activity of the HPA (Räikkönen et al., 2011; Beijers et al., 2014). Thus, cortisol has been considered as an important mediator of the effects of maternal stress on the developing brain of the fetus (review: Entringer et al., 2015). Specifically, this effect is assumed to be regulated by the placental enzyme 11 beta-hydroxysteroid dehydrogenase type 2 (11beta-HSD2) which converts cortisol in its inactive form cortisone (Beitins et al., 1973; Brown et al., 1996). Importantly, some proportion of active maternal cortisol passes through the placenta into the fetal compartment. In line with this assumption, adverse intrauterine conditions such as maternal anxiety (O'Donnell et al., 2012), severe infection (Johnstone et al., 2005), or alcohol exposure (Liang et al., 2011) have been found to co-occur with a down-regulation of placental 11beta-HSD2 activity. Another mechanism that may regulate the fetal programming effects of elevations in maternal cortisol is increased production of placental corticotrophin-releasing hormone (CRH), which, in turn, may affect the fetal HPA axis and the biosynthesis of adrenal steroids (review: Entringer et al., 2015).

Previous evidence found several parental risk factors that may affect long-term regulation of the body's stress response systems in the offspring, e.g., low socioeconomic status (Essex et al., 2002; Evans and Kim, 2007) or maternal psychopathology (Gentile, 2017). Specifically, changes in maternal cortisol regulation due to (traumatic) stress, anxiety, or depressiveness have been shown to result in altered fetal cortisol levels as measured in saliva (Yehuda et al., 2005; Brennan et al., 2008; Grant et al., 2009; Davis et al., 2011) and urine (Yehuda et al., 2002; Diego et al., 2004). However, the reported relationships between various measures of prenatal maternal stress and offspring cortisol are complex and heterogeneous (Van den Bergh et al., 2017) which may be partly the result of the use of short-term cortisol measures as mentioned earlier. Recently, research using HCC analysis among newborns is emerging (e.g., Yamada et al., 2007; Hoffman et al., 2017) highlighting the unique potential of neonatal HCC to reflect intrauterine glucocorticoid regulation. Specifically, it has been shown that cortisol levels of neonatal hair which was taken at birth mainly reflect the third trimester increase of maternal cortisol (Hollanders et al., 2017).

Studies examining HCC in infants found associations to maternal HCC during pregnancy, supporting the notion of a transgenerational transmission of alterations of the long-term activity of the HPA axis (Karlén et al., 2013; Romero-Gonzalez et al., 2018). As mentioned above, recent studies found close links between trauma exposure across the lifespan and long-term regulation of HPA axis activity (Steudte-Schmiedgen et al., 2016; Pervanidou et al., 2017). Interestingly, initial cross-sectional HCC research supports the notion of a relationship between lifetime trauma exposure, childhood abuse, and HCC during pregnancy (Schreier et al., 2015, 2016; Swales et al., 2018). A prospective study further confirmed associations between maternal lifetime trauma history, HCC reflecting the third trimester of pregnancy, and subsequent infant negative affectivity at the age of 6 month (Bosquet Enlow et al., 2017). This is commensurate with study data observing an association between maternal lifetime trauma exposure and increased HCC in older children (i.e., at the age of three and four), albeit this relationship was not found in infants under the age of two (Slopen et al., 2018). These promising findings are in line with the assumption of an intergenerational transmission of maternal childhood maltreatment and necessitate further prospective investigations (Buss et al., 2017).

Not only intrauterine but also postpartum factors can affect long-term activity of the HPA axis of children as measured by HCC analysis. Research about the role of parent-infant interaction is slowly emerging. For example, a study found that mothers with increased HCC were more intrusive and showed lower positive engagement synchrony with their offspring 6 months after delivery (Tarullo et al., 2017). This corresponds with a study showing an association between mother's parenting stress and depressiveness at 4 weeks postpartum and higher HCC in their infants at the age of one, which, in turn, were related to pronounced socioemotional problems (Palmer et al., 2013). Thus, both intrauterine and postpartum influences on the infant HPA axis can manifest in infant health and behavior, e.g., a difficult temperament or regulatory problems that can precede later psychopathology and impair further development of the child (Gunnar and Donzella, 2002; Hemmi et al., 2011; Davis and Sandman, 2012; Stein et al., 2014; Petzoldt et al., 2016). Still, evidence regarding the relations between maternal self-reported distress, activity of the HPA axis, and infant outcomes is rare, inconsistent, and mainly stems from studies assessing short-term cortisol (e.g., in saliva: Bosquet Enlow et al., 2017; Van den Bergh et al., 2017). Hence, there is need of further investigations to detect how maternal HCC are reflected in long-term changes of health and behavior problems and possibly underlying biological stress reactions of the child indicated by neonatal HCC.

Moreover, it is of significance whether psychological intervention can support a normalization of HPA axis dysregulation in both mother and child. Over the past years, evidence emerged supporting the notion that effective psychotherapy may improve this kind of dysregulations in patients suffering from posttraumatic stress disorder. Specifically, cortisol levels were found to increase in responders to psychotherapy while those of non-responders decreased (Olff et al., 2007; Yehuda et al., 2009, 2014), albeit with some inconsistency in findings (review and meta-analysis: Gerardi et al., 2010; Pacella et al., 2014; Schumacher et al., 2018). Further, evidence has accumulated in support of the potential of pre-treatment hormone concentrations as predictors of successful psychotherapeutic outcome in patients with posttraumatic stress disorder (review: Colvonen et al., 2017) and patients with affective and/or anxiety disorders (Fischer et al., 2018). Regarding postpartum mental disorders, treatments combining psychological, psychopharmacological, and interactional components in a mother-baby unit (MBU) are found to contribute to lower psychopathology (review: Connellan et al., 2017). So far, no study has investigated the role of long-term endocrine correlates as predictor and correlate of successful clinical outcome of such a treatment among mothers and their infants. If there are alterations of the long-term activity of the HPA axis going along with the therapy, considering the linkage between maternal and infant HPA axis, it can be reasonably assumed that not only maternal health but also infant health and behavior and its endocrine correlates may improve within treatment of postpartum mental disorders.

As mentioned above, the involvement of fathers with their children is slowly increasing (Barker and Pawlak, 2011; Bundesministerium für Familie Senioren Frauen und Jugend, 2017a). Still, the role of fathers and partners related to the mother has been widely neglected in previous studies. In particular, there is a lack of evidence with regard to the mechanisms through which the partner may have an impact on the child (Barker et al., 2017). Emerging research suggests that, in accordance to the evidence regarding mothers, early father-infant-interaction and parenting affect child development and behavior (Ramchandani et al., 2013; Parfitt et al., 2014). Consistent with this, psychopathology of the father has detrimental effects on parenting (review: Wilson and Durbin, 2010) and (maybe in turn) on child outcomes (Ramchandani et al., 2005; Sweeney and MacBeth, 2016). Regarding endocrinological aspects, recent studies indicate that couples' mental health and physiological states (especially cortisol) are associated with one another, which is assumed to have implications for health and functioning as a couple, e.g., role distribution or partnership satisfaction (Timmons et al., 2015), which, in turn, may have a direct or indirect impact on child outcomes (Hanington et al., 2012; Parfitt et al., 2014).

In conclusion, the impact of long-term effects of stress, e.g., caused by precarious working conditions or psychosocial work stress, on mental and somatic health of the whole family warrants more research. In particular, previous studies were limited to self-reported measures or short-term cortisol assessment methods (e.g., saliva or urine). Consequently, more detailed information about the HPA axis linkage between parents and in parent-child dyads is needed to examine the pathways of transgenerational transmission of long-term regulation of the HPA axis and possible approaches for intervention.

To close this gap, the current cohort study called Dresden Study on Parenting, Work, and Mental Health (**DREAM**; “**DR**esdner Studie zu **E**lternschaft, **A**rbeit und **M**entaler Gesundheit”) together with its sub-studies DREAM_HAIR_ and DREAM_TALK_ combining quantitative questionnaires, qualitative interviews, and long-term endocrine correlates aims to examine the impact of parental work participation, role distributions, and stress factors on family health longitudinally, i.e., during the course from late pregnancy to 2 years postpartum.

**Figure 1:**
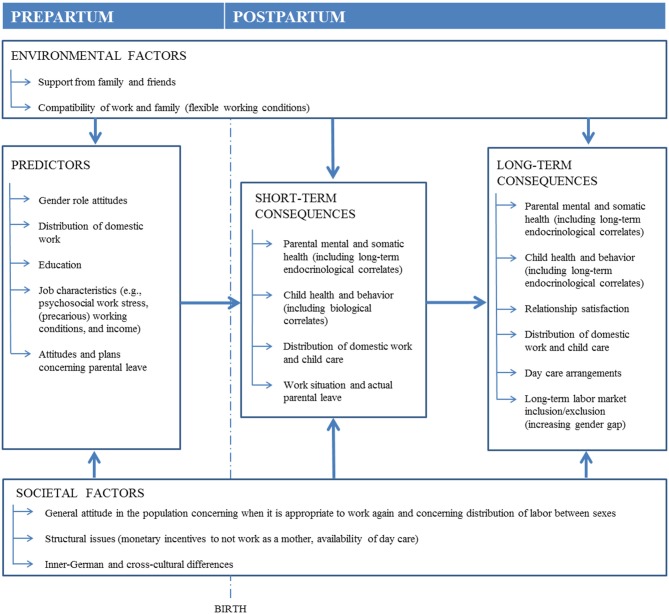
Simplified theoretical model upon which the DREAM study is based.

Based on the theoretical model shown in Figure 1, the following main questions will be investigated:

- How do mothers' and their partners' work participation and role distribution regarding domestic work, child care, and paid employment change in the course from pregnancy to 2 years postpartum; in particular, will there be a shift toward more traditional gender roles (DREAM and DREAM_TALK_)?
- How are mothers' and their partners' mental and somatic health as well as child health and behavior influenced by their work participation over time and is a potential association influenced by confounding or moderating factors such as precarious working conditions and psychosocial work stress (DREAM and DREAM_TALK_)?
- How does mothers' and their partners' mental health affect child health and behavior cross-sectionally and longitudinally (DREAM)?
- How are HCC inter-related between the family members (mother, father, index child) cross-sectionally and longitudinally (DREAM_HAIR_)?
- Are HCC predicted by stress factors (including precarious working conditions and psychosocial work stress)? How do HCC relate to mental health and behavioral outcomes of each family member cross-sectionally and longitudinally (DREAM_HAIR_)?
- Regarding a clinical sample, are maternal mental disorders and long-term HCC of mothers (and partners if available) at admission to the MBU associated with behavioral outcomes and HCC of the child and how does this change during the course of psychotherapeutic treatment (DREAM_MBU_)?

With its multi-method approach addressing both parents and child, this study is unique in the research on the interplay of work participation, role distribution, and stress factors and its impact on perinatal outcome and mental and somatic health of the whole family. Considering the complex relationships between prenatal and postnatal psychological distress and long-term cortisol secretion in parents and their offspring, this study will make a significant contribution to the existing literature.

## Methods and analyses

### Overall Design and Procedure

The DREAM study is a prospective cohort study targeting a total community sample of *N* = 4,000 individuals, i.e., 2,000 couples, expecting a child in and around Dresden, Germany. Inclusion criteria are a current pregnancy, being a resident in the mentioned area, and sufficient German skills to complete the study questionnaires. In order to get a comprehensive picture of (becoming) parents in the area, there was no exclusion of comparatively rare groups, e.g., multiple pregnancies, couples of the same sex, and single persons. Since June 2017, pregnant women [subsequently referred to as (expectant) mothers], and the male or female partners they are currently involved with in a long-term relationship are recruited during pregnancy mainly in obstetrical clinics and midwife practices, with an interim sample of *N* = 1,410 participants who have completed the first questionnaire (T1) by the end of September 2018 (recruitment ongoing). The DREAM study has been approved by the Ethics Committee of the Faculty of Medicine of the Technische Universität Dresden (No: EK 278062015).

**Figure 2:**
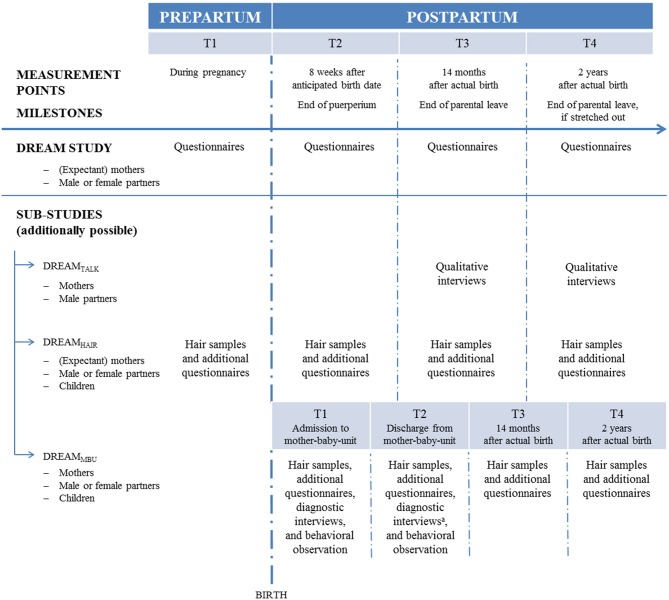
Assessment waves of the DREAM study and its sub-studies DREAM_TALK_ (qualitative interview sub-study), DREAM_HAIR_ (long-term endocrinological sub-study), DREAM_MBU_ (clinical sub-study). Regarding the community sample, T1 during pregnancy, T2 8 weeks after anticipated birth date, T3 14 months, and T4 2 years after actual birth date (prolongation into middle childhood planned). Regarding the clinical sample, T1 at admission and T2 at discharge (hair samples will be taken 2 months after discharge) from mother-baby-unit, T3 14 months (will only be omitted if the sampling point at discharge from mother-baby-unit is not too close, i.e., 14 months postpartum ± 2 months), T4 at 2 years after actual birth date. ^a^At discharge, diagnostic interviews will only be omitted regarding children.

Following milestones for young German families, in the basic DREAM study there are four measurement points (Figure 2) starting with T1 during pregnancy and three postpartum assessment waves, i.e., T2 at 8 weeks after the anticipated birth date when puerperium is over, T3 at 14 months, and T4 at 2 years after the actual birth date. T3 and T4 go along with German parental allowance because T3 is at the time the parental allowance is over (if the couple took the full allowance and both parents were on parental leave for at least 2 months) and T4 is at the time the stretched out parental allowance (for families who worked part-time for a while) ends. A prolonged investigation of families into middle childhood is planned. The DREAM study is conducted in a larger city in East Germany where both maternal work participation and day care are a more frequent practice than in West Germany (Bundesministerium für Familie Senioren Frauen und Jugend, 2017a). Therefore, the DREAM study cooperates with a West German study called the Bremen Initiative to Foster Early Childhood Development (“BRISE”; Bremer Initiative zur Stärkung frühkindlicher Entwicklung). Moreover, data of the DREAM study will be compared to data of the Norwegian Akershus Birth Cohort (ABC study). This cross-cultural comparison to a country that is more progressive regarding gender considerations (Barker and Pawlak, 2011) will complement the results about the role of work participation for family health. For this purpose, measurement points and many instruments are matched.

Applying a multi-method approach, the DREAM study is complemented by two sub-studies: DREAM_HAIR_ (analysis of HCC and other endogenous steroid hormones) and DREAM_TALK_ (qualitative interviews regarding gender role values and distribution of domestic work, child care, and paid employment). Participants meeting inclusion criteria for the particular sub-study are contacted again to be invited to participate in the sub-study. Due to the focus of this paper, the basic DREAM study and the sub-study DREAM_HAIR_ are presented in detail.

### Sub-studies

#### DREAM_HAIR_ and DREAM_MBU_

The long-term endocrinological sub-study DREAM_HAIR_ aims to examine the complex relationships between psychological distress and long-term steroid hormone levels in parent-child dyads from pregnancy to 2 years postpartum (prolongation into middle childhood planned). This sub-study consists of two branches: a community based approach (DREAM_HAIR_, already started) and a clinical approach (DREAM_MBU_, starting soon). Within the community branch, the community sample stems from the basic DREAM study. Within the clinical branch, a clinical sample of mothers with postpartum mental disorders, their partners (if available), and their children are recruited from a mother-baby day clinic. Data of the community and clinical sample will be combined for long-term analyses.

Recruitment for the community branch of the sub-study DREAM_HAIR_ began 5 months after the start of the basic DREAM study. For this sub-study, all families whose expected delivery date is at least 4 weeks in the future are checked for inclusion criteria. Inclusion criteria for parents comprise a minimal hair length of 2 cm, no hair loss or baldness, no severe physical disease (e.g., cancer or adrenocortical dysfunction) over the last 5 years, and no use of glucocorticoid containing medication for the last 4 months. Regarding children, there are no particular reasons for exclusion other than no availability of hair (without a minimal hair length). Potentially confounding variables (e.g., medication, diseases) are assessed and used for further analyses. Inclusion criteria will be checked at every measurement point. If participants are excluded at a single measurement point due to temporary reasons, e.g., medication or too short hair, or they miss an assessment wave due to organizational reasons, they will be asked again at the next measurement point. Participants can join this sub-study as a couple or alone as long as each parent who has child custody provides her/his consent that child hair is also allowed to be taken.

After being enrolled into the basic DREAM study, hair samples of the eligible expectant mothers, their partners (i.e., *N* = 152 participants, i.e., 88 families, at T1 by the end of September 2018) and their children, are examined for HCC and other endogenous steroid hormones at four measurement points from pregnancy up to 2 years postpartum (Figure 2; prolongation into middle childhood planned). Regarding the parents, T1_DREAMHAIR_ starts 4 to 6 weeks prior to the anticipated birth date which reflects HCC integrated during late pregnancy. Regarding the children, T1_DREAMHAIR_ hair samples are taken soon after birth to gain information on intrauterine glucocorticoid regulation (Hollanders et al., 2017). The other measurement points (T2_DREAMHAIR_, T3_DREAMHAIR_, and T4_DREAMHAIR_) are equivalent to the basic DREAM study.

At the respective first measurement point, hair samples of community sample will be taken at their home or at the lab as preferred by the participants while hair samples of the clinical sample will be taken at the lab in any case. In both samples, the first hair sample is taken by trained staff. In order to self-administer hair samples at the follow-ups, participants get a video teaching how to take own and child hair samples themselves or with help of their partner or a friend. At the follow-ups, participants get a reminder when the next hair samples are due. As we are aware that this sub-study requires some more commitment by the participants compared to the basic questionnaire study, we put emphasis on personal contact to the participants. For instance, trained staff assists them with taking own or child hair sample if needed.

For the clinical branch (DREAM_MBU_), recruitment of a clinical sample of approximately *N* = 70 mothers with postpartum mental disorders who are treated in the day-care MBU at the Institute and Outpatient Clinics of Psychotherapy and Psychosomatic Medicine at the Technische Universität Dresden is planned starting by the year 2019 (DREAM_MBU_). In this MBU, mothers with severe postpartum mental disorders (e.g., depressive disorders, anxiety disorders, obsessive-compulsive disorders, and personality disorders) are treated together with their child (0–12 months old, mean child age at admission is 23.3 weeks (*SD* = 13.1) for averagely 8.5 weeks (*SD* = 3.1). Based on a treatment concept that is state of the art for postpartum mental disorders (Wortmann-Fleischer et al., 2012), patients get a treatment consisting of both mother focused (e.g., disorder specific psychotherapy, psychopharmacotherapy) and mother-infant-interaction focused components (e.g., mother group treatment, video-interaction-therapy, baby massage and handling, sensitivity training) conducted by a multi-professional team. Partners and other family members are involved in the treatment if possible.

For the clinical sub-study DREAM_MBU_, patients will be asked for written informed consent to take part in the study within the first week after admission. Their children and (if available) partners will also be included. As shown in Figure 2, hair samples of all participants will be taken four times: once at admission (T1_DREAMMBU_) to measure the steroid exposition during the 2 months prior to treatment and a second time (T2_DREAMMBU_) 2 months after discharge from MBU to assess HCC during the 2 months after treatment. Further, to compare the data of the clinical sample to the data of the community sample at T3_DREAMHAIR_ and T4_DREAMHAIR_ (14 and 24 months postpartum), a third (T3_DREAMMBU_), and fourth (T4_DREAMMBU_) hair sample of the clinical population will be taken [if the sampling point at discharge (T2_DREAMMBU_) is very close to T3_DREAMHAIR_ (14 ± 2 months), one sampling point will be omitted].

In the clinical part of the sub-study DREAM_MBU_, patients and their partners will also go through the routine assessment of the treatment effects (Table 1) using a comprehensive assessment battery at admission and discharge. At admission, patients will be interviewed using the Structured Clinical Interview for DSM-IV (SCID I and SCID II; Wittchen et al., 1997). Both at admission and discharge, the Diagnostic Interview for Mental Disorders in Babies showing good to very good inter-rater reliability (Baby-DIPS; Popp et al., 2016), a semi-standardized observation of maternal sensitivity toward their baby's signals (Galeris, 2016; validation pending), and additional questionnaires regarding the maternal psychopathology and parenting as well as infant temperament, regulation disorders, and behavior will be obtained. There is an overlap between the MBU assessment and the basic DREAM assessment regarding all relevant measures.

**Table 1:** Constructs and instruments in the basic DREAM study.

		**Community sample (DREAM)**				**Clinical sample (DREAM**_**MBU**_**)**			
**Constructs**	**Instruments**	**T1**	**T2**	**T3**	**T4**	**T1**	**T2**	**T3**	**T4**
**SOCIODEMOGRAPHIC AND SOCIOECONOMIC FACTORS**									
Nationality and mother tongue	Questions derived from the German National Cohort (German National Cohort Consortium, 2014)	x				x			
Education	Questions derived from the German National Cohort (German National Cohort Consortium, 2014)	x				x			
Marital status	Questions derived from the Socio-Economic Panel (SOEP; TNS Infratest Sozialforschung, 2016) and self-generated questions	x	x	x	x	x	x	x	x
Children and former pregnancies	Questions derived from the BRISE study based on the BabyCare Project (Friese and Kirschner, 2003)	x	x			x	x		
Housing	Questions derived from the German National Cohort (German National Cohort Consortium, 2014)	x	x	x	x	x	x	x	x
**WORK-RELATED FACTORS**									
Working hours and professional group	Questions derived from the German National Cohort (German National Cohort Consortium, 2014)	x		x	x	x	x	x	x
Job satisfaction and job burden	Questions derived from the BRISE study based on the BabyCare Project (Friese and Kirschner, 2003) and the Exploration Questionnaire for Identification of Differential Learning Paths in the Social Development in Toddler Age (“Explorationsfragebogen zur Identifikation differentieller Lernwege in den ersten beiden Lebensjahren”, IDL 0-2; Petermann et al., 2002)	x		x	x	x	x	x	x
Sick leave and adaption of work situation in pregnancy	Questions derived from the ABC study (e.g., Dørheim et al., 2013)	x		x	x	x	x	x	x
Shift work	Questions derived from the BRISE study based on the BabyCare Project (Friese and Kirschner, 2003)	x		x	x				
Precariousness	Employment Precariousness Scale–Revised (EPRES; Vives et al., 2015)	x		x	x				
Psychosocial work stress	Effort-Reward Imbalance Questionnaire (ERI; Siegrist, 1996; Rödel et al., 2004)	x		x	x				
Work-privacy conflict	One scale (work-privacy conflict) of the Copenhagen Psychosocial Questionnaire (COPSOQ; Kristensen et al., 2005; Nübling et al., 2005)	x		x	x				
Plans and actual parental leave	Self-generated questions	x	x	x	x	x	x	x	x
Satisfaction with distribution of parental leave	Self-generated questions			x	x			x	x
**DISTRIBUTION OF DOMESTIC WORK AND CHILD CARE**									
Attitudes toward distribution of domestic work	Nine scales (effective communication about domestic labor, ministering to family needs, support of wage work, responsive to personal needs, avoiding conflict, coprovider orientation, valuing homemaking, standards, women's ultimate accountability) of the Orientation Toward Domestic Labor Questionnaire (ODL-Q; Hawkins et al., 1998)	x		x	x				
Distribution of domestic work and child care	Questions derived from the ABC study	x	x	x	x				
Time spent for domestic work and child care	Questions derived from the 1997 National Study of the Changing Workforce (Hall and MacDermid, 2009)			x	x				
**SOMATIC FACTORS**									
Current and former somatic health	Questions derived from the ABC study (e.g., Garthus-Niegel et al., 2018; Junge et al., 2018)	x	x	x	x	x	x	x	x
Exercise and physical activity	Questions derived from the ABC study generated by health professionals (e.g., Gjestland et al., 2013)	x		x	x	x	x	x	x
Health-related quality of life	Short-Form Health Survey (SF-8; Ware et al., 2001; Ellert et al., 2005)				x				x
Drugs	Questions derived from the ABC study generated by health professionals (e.g., Nordeng et al., 2012)	x		x	x	x	x	x	x
Smoking	Slightly modified questions derived from the German National Cohort (German National Cohort Consortium, 2014)	x	x	x	x	x	x	x	x
Alcohol	Questions derived from the German National Cohort (German National Cohort Consortium, 2014)	x	x	x	x	x	x	x	x
**MENTAL FACTORS**									
Current and former mental disorders and treatments	Self-generated questions Structured Clinical Interview for DSM-IV (SCID I and SCID II; Wittchen et al., 1997)		x	x	x	xx	x	x	x
Use of early help	Self-generated questions		x			x			
Symptoms of depression	Edinburgh Postnatal Depression Scale (EPDS; Cox et al., 1987; Bergant et al., 1998)	x	x	x	x	x	x	x	x
Psychopathological symptoms	Four scales (somatization, obsessiveness, anxiety and anger/hostility) of the Symptom Check List–Revised (Derogatis, 1977; Franke and Derogatis, 2002) Brief symptom inventory (BSI; Derogatis, 1993; Franke, 2000)	x	x	x	x	x	x	x	x
Current and former critical life events	Questions derived from the BRISE study based on the Avon Longitudinal Study of Parents and Children (ALSPAC; Thomson et al., 2014)		x	x		x	x	x	
Current and former posttraumatic stress reactions	Posttraumatic Diagnostic Scale (PDS; Ehlers et al., 1996; Foa et al., 1997)	x^c^		x^c^	x^c^	x	x	x	x
Adverse childhood experiences	Childhood Trauma Questionnaire (CTQ; Bernstein and Fink, 1998; Wingenfeld et al., 2010)	x^c^				x			
Intradyadic stress and extradyadic stress from daily hassles	Multidimensional Stress Questionnaire for Couples (“Multidimensionaler Stressfragebogen für Paare”, MDSP; Bodenmann, 2007)				x				x
**RELATIONSHIP FACTORS**									
Relationship satisfaction	Short version of the Partnership Questionnaire (“Kurzform des Partnerschaftsfragebogens,” PFB-K; Kliem et al., 2012)	x	x	x	x	x	x	x	x
Social support	Short version of the Social Support Questionnaire (“Fragebogen zur Sozialen Unterstützung,” F-SozU-14; Fydrich et al., 2009)	x		x	x	x	x	x	x
**PREGNANCY AND BIRTH-RELATED FACTORS**									
Fear of childbirth^a^	Fear of Birth Scale (FOBS; Haines et al., 2011)	x							
General information and complications during pregnancy and birth^a^	Maternity records (“Mutterpass”; Gemeinsamer Bundesausschuss, 2015), questions derived from the BRISE study, and self-generated questions		x			x			
Birth experience^b^	Salmon's Item List (SIL; Stadlmayr et al., 2001)		x			x			
Overall birth experience^b^	Question derived from the ABC study (e.g., Garthus-Niegel et al., 2014)		x			x			
Fear for oneself/mother and child during birth^b^	Self-generated questions based on the ABC study (e.g., Garthus-Niegel et al., 2013)		x			x			
Fear of a prospective birth^b^	Question derived from the ABC study		x			x			
Birth-related post-traumatic stress reactions	Impact of Event Scale–Revised (IES-R; Weiss and Marmar, 1996; Maercker and Schützwohl, 1998)		x			x			
**CHILD-RELATED FACTORS**									
Breastfeeding	Self-generated questions based on recommendations of the World Health Organization (World Health Organization, 2009)		x	x	x	x	x	x	x
Parent-to-infant-bonding	Postpartum Bonding Questionnaire (PBQ; Brockington et al., 2001; Reck et al., 2006)		x	x	x	x	x	x	x
Sensitivity toward baby's signals	Semi-standardized observation of maternal sensitivity (global score and six subscales) toward baby's signals (Galeris, 2016)					x	x		
Parenting sense of competence	Parenting Sense of Competence Scale (“Fragebogen zum Kompetenzgefühl von Eltern,” FKE; Gibaud-Wattston and Wandersman, 1978, as cited in Johnston and Mash, 1989; Miller, 2001)					x	x	x	x
Parenting stress	Parenting Stress Index (“Eltern-Belastungs-Inventar,” EBI; Abidin, 1995; Tröster, 2011)					x	x	x	x
Infant regulation disorders	Structured Diagnostic Interview for Regulatory Problems in Infancy (“Diagnostisches Interview bei psychischen Störungen im Säuglings- und Kleinkindalter,” Baby-DIPS; Popp et al., 2016)					x	x		
Child health	Medical records (“Kinderuntersuchungsheft”; Gemeinsamer Bundesausschuss, 2016) and questions derived from the ABC study		x	x	x	x	x	x	x
Child development	Five scales (communication, gross motor, fine motor, problem solving, personal-social) of the 14 and 24 month version of the Ages and Stages Questionnaire-3 (ASQ-3; Squires and Bricker, 2009)			x	x			x	x
Child temperament	One scale (fussy/difficult scale) of the Infant Characteristics Questionnaire (ICQ; Bates et al., 1979)		x						
	Infant Behavior Questionnaire (IBQ; Rothbart, 1981; Pauli-Pott et al., 1999)					x			
Child care	Questions derived from the Socio-Economic Panel (SOEP; TNS Infratest Sozialforschung, 2016)			x	x	x	x	x	x
**PERSONALITY**	Big Five Inventory–SOEP (BFI-S; Schupp and Gerlitz, 2008)		x						
**METACOGNITION**	Metacognition Questionnaire–Short version (MFK-30; Wells and Cartwright-Hatton, 2004; Arndt et al., 2011)		x						

*Regarding community sample (DREAM), T1 during pregnancy, T2 8 weeks after anticipated birth date, T3 14 months, and T4 2 years after actual birth date (prolongation into middle childhood planned). Regarding clinical sample (DREAM_MBU_), T1 at admission and T2 at discharge (hair samples will be taken 2 months after discharge) from mother-baby-unit, T3 14 months (will only be omitted if the sampling point at discharge from mother-baby-unit is not too close, i.e., 14 months postpartum ± 2 months), T4 at 2 years after actual birth date*.

a *Only for expectant mothers*.

b *Male and female partners only asked when they have attended birth*.

c *Only for DREAM_HAIR_*.

#### DREAM_TALK_

The qualitative DREAM_TALK_ sub-study will start during summer 2019. A subsample targeting approximately *N* = 40 heterosexual couples, i.e., women and men engaged in a long-term relationship and living in the same household, will be interviewed on their gender role values and attitudes as well as thoughts about distribution of domestic work, child care, and paid employment. These attitudes will be compared to the actual situation. Additionally, both partners' satisfaction regarding their relationship and role distribution as well as how they explain a potential equality gap will be assessed. Results will be analyzed in relation to health-related outcomes measured in the basic DREAM study.

Based on a synthesis of previous studies on different task distributions (Crouter and Manke, 1997; Hall and MacDermid, 2009; Farrokhzad et al., 2010; Helms et al., 2010; Masterson and Hoobler, 2015; Bundesministerium für Familie Senioren Frauen und Jugend, 2017b), couples will be categorized into one of four groups by a cut-off threshold of hours spent on domestic work, child care, and paid employment to compare those groups: progressive couples sharing domestic work, child care, and paid work equally; traditional couples with women doing most of the domestic work and child care and men doing most of the paid work; as well as two interjacent groups.

As shown in Figure 2, qualitative interviews, specifically problem-centered interviews (PCI) following Witzel and Reiter (2012), will be carried out a few weeks after T3 and T4 of the basic DREAM study. This is at the time the regular and the stretched out parental allowance run out. The interviews will be analyzed using qualitative content analysis by Mayring (2010). Categories for the collected interview data will be established, yielding interpretations of the participants' answers in a replicable and systematic way.

Taking into account the participants' wishes, the interviews will take place at the couples' home or at the lab. Trained doctoral students following interview guidelines will conduct them. The project team aims to achieve a personal connection and identification with the study among the participants in order to keep attrition low.

### Materials

The DREAM study is characterized by a multi-method approach combining quantitative questionnaires (basic DREAM study), long-term endocrine correlates (DREAM_HAIR_), and qualitative interviews (DREAM_TALK_). Study data are collected and managed using Research Electronic Data Capture (REDCap), a secure, web-based application designed to support data capture for research studies, hosted at the “Koordinierungszentrum für Klinische Studien” at the Faculty of Medicine of the Technische Universität Dresden, Germany (Harris et al., 2009). In this article, we focus on the questionnaire data and analysis of HCC.

#### Questionnaires

The DREAM study comprises several questionnaires as presented in Table 1. Standardized and validated instruments with good psychometric properties were preferably used. For example, the German version of the Edinburgh Postnatal Depression Scale (EPDS; Cox et al., 1987; Bergant et al., 1998), a well-established, reliable, and valid instrument was obtained to measure prepartum and postpartum symptoms of depression, while the Effort-Reward Imbalance Questionnaire (ERI; Siegrist, 1996; Rödel et al., 2004) being characterized with satisfactory psychometric characteristics was used to assess psychosocial work stress. Where possible, instruments were chosen in agreement with the BRISE project team, the German National Cohort (German National Cohort Consortium, 2014), or the Norwegian ABC study to allow intra- and intercultural comparisons. If no German version of a questionnaire existed, the English or Norwegian version was translated into German and back-translated by a native speaker (validation still pending).

In the sub-studies, relevant questionnaires were added. In DREAM_HAIR_, participants complete a self-generated questionnaire about hair-related characteristics, e.g., washes per week, curls, or hair treatments (as described in Stalder et al., 2014). Additionally, hair analyses were complemented by trauma specific questionnaires (Table 1). Specifically, the checklist of the Posttraumatic Diagnostic Scale (PDS; Ehlers et al., 1996; Foa et al., 1997) to assess the nature and presence of the most upsetting traumatic event (A1 and A2 criteria of DSM-IV, time since occurrence) as well as the Childhood Trauma Questionnaire (CTQ; Bernstein and Fink, 1998; Wingenfeld et al., 2010) to measure the severity of childhood maltreatment were added. The German adaptions of both questionnaires have been found to be reliable and valid instruments similar to their English original versions (Griesel et al., 2006;Wingenfeld et al., 2010).

In order to attain best possible response and retention rates, participants can decide whether to complete online or paper-pencil versions of the questionnaires. By the end of September 2018, *N* = 1,410 participants, i.e., expectant mothers and their partners, have given consent to join the study and completed the first questionnaire which is necessary to take part in the follow-ups. Almost half of them (47.6%; *n* = 671) decided for the paper-pencil procedure with a somewhat larger ratio of expectant mothers (50.7%; *n* = 408) compared to their partners (43.5%; *n* = 259). After completing T1, the participants receive the subsequent questionnaires and reminders in the desired manner automatically, even if they missed one of the measurement points. To keep attrition low, participants get incentives together with the follow-up questionnaires at every measurement point, e.g., rompers, bibs, or books, to encourage them to answer in time. In case of breakup, participants will be contacted separately, so this is no reason to drop out.

#### Hair Cortisol Concentrations (HCC) Analyses

For analyses of HCC as part of the sub-study DREAM_HAIR_, a hair strand of a diameter of ~3 mm is taken scalp-near from a posterior vertex position. HCC will be determined in the 2 cm hair segment most proximal to the scalp. If permitted by the individuals' hair length, the next 2 cm of hair will additionally be analyzed. Based on a hair growth rate of ~1 cm/month (Wennig, 2000), these hair segments are assumed to represent integrated, cumulated cortisol levels over the 2 month or, if permitted, 4 month period prior to hair sampling. Hair samples will be stored in aluminum foil and sent to the Institute of Biological Psychology at the Faculty of Psychology of the Technische Universität Dresden. Hair analyses will be conducted following the liquid chromatography-tandem mass spectrometry (LC-MS/MS) protocol which is characterized by very good sensitivity, specificity, and reliability (Gao et al., 2013). Besides cortisol, the LC-MS/MS protocol allows the quantitative analysis of other endogenous steroid hormones, i.e., cortisone, testosterone, progesterone, corticosterone, dehydroepiandrosterone (DHEA), and androstenedione that may serve as important mediators in the development of psychopathology or psychological distress (Gao et al., 2013).

### Sample

In this section, the interim samples of the basic DREAM study and the sub-study DREAM_HAIR_ (community branch) by the end of September 2018 will be presented. The participants get no payment for their participation but incentives at every measurement point, e.g., rompers or bibs, as mentioned earlier. Additionally, a lottery is established so two participants may win vouchers and gifts every month.

#### Interim Sample and Flow Chart of DREAM

**Figure 3:**
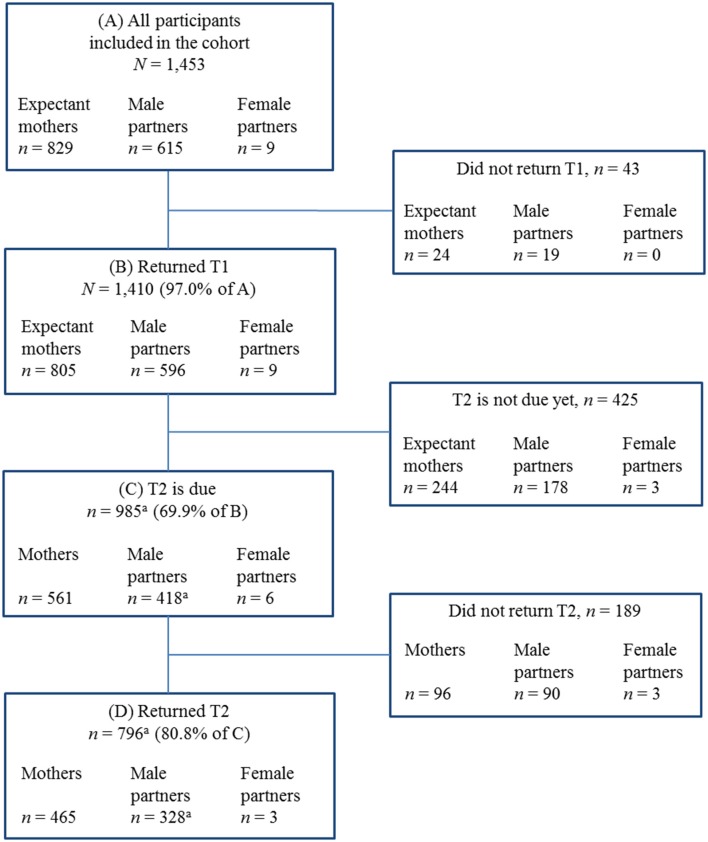
Flow chart of the DREAM study. Notes: T1 during pregnancy, T2 8 weeks after anticipated birth date. Data from end of September 2018 (data collection is not finished yet, recruitment ongoing). Further future assessment waves (not due yet): T3 14 months and T4 2 years after actual birth date (prolongation into middle childhood planned). ^a^Male partners' sample includes one male partner who did not return T1 but returned T2.

The DREAM study addresses expectant mothers and their male or female partners in or around Dresden, Germany. The flow chart of the DREAM study is presented in Figure 3.

*N* = 1,453 expectant mothers and their partners have provided their written consent to participate from June 2017 to the end of September 2018 (recruitment still ongoing). Of those having provided consent, the vast majority of *N* = 1,410 (97.0%) participants completed the first questionnaire (T1) resulting in a sample of *n* = 805 expectant mothers (57.1%) and *n* = 596 male partners (42.3%), plus *n* = 9 female partners (0.6%). At T1, most of the participants joined the study as a couple (81.3%; *n* = 1,146 people, i.e., 573 couples), but in some cases an expectant mother participated without a partner (16.4%; *n* = 232) and a few partners participated without the expectant mother (2.3%; *n* = 32).

As shown in Table 2, participants who returned T1 were recruited in obstetrical clinics in Dresden and the surrounding area (79.5%; *n* = 1,120), i.e., at information events and tours of the delivery rooms, scheduled birth registration done by midwives, and at the inpatient prenatal treatment ward. Further participants were included from a freestanding birthing center (4.1%; *n* = 58), at birth preparation courses in midwife practices (6.7%; *n* = 95), and at the early care of the city of Dresden (“Frühe Hilfen”; 0.1%; *n* = 2). A certain number of participants (4.4%; *n* = 62) were recruited via other places, i.e., mainly via announcements in gynecological outpatient settings, child-related stores, websites, newspapers, and magazines of health insurances, or through personal contact. Also, some participants (5.2%; *n* = 73) could not be assigned to a specific recruitment way.

**Table 2:** Total sample who returned T1 (*N* = 1,410) according to recruitment way.

**Recruitment way**	**Sample (*****N*** **= 1,410)**	
	*n*	*(%)*
Obstetrical clinics (*N* = 8): information evenings and tours of the delivery rooms	1,049	(74.4)
- *there of Dresden* (*N* = 5)	961	(91.6)
- *there of surrounding area* (*N* = 3)	88	(8.4)
Obstetrical clinic (*N* = 1): scheduled birth registration	38	(2.7)
Obstetrical clinic (*N* = 1): inpatient prenatal treatment ward	33	(2.4)
Freestanding birthing center (*N* = 1): information evenings	58	(4.1)
Midwife practices (*N* = 12): birth preparation courses	95	(6.7)
City of Dresden: early care (“Frühe Hilfen”)	2	(0.1)
Other places	62	(4.4)
Unknown places	73	(5.2)

The number of different people approached was varying between the locations. For example, 25–100% of the attendees of the birth preparation courses knew the study already as they often visited at least one of the clinics' information evenings as well. In sum, there was personal contact to *N* = 9,477 persons (i.e., expectant mothers and their male or female partners). Given that *n* = 1,348 participants provided written informed consent (not including those who were recruited via other places) this would indicate a response rate of 14.2%. However, the true response rate is considerably higher but hard to calculate exactly because a much smaller number was eligible due to multiple visits.

By the end of September 2018, 80.8% (*n* = 796) of those participants T2 was due for (*n* = 985) have sent back the second questionnaire which is 8 weeks after the expected delivery date. The retention rate from T1 to T2 is best for mothers (82.9%; *n* = 465) followed by male (78.5%; *n* = 328) and female partners (50.0%; *n* = 3). As some participants do not send back the follow-ups in time but with some delay, we expect the retention rate to increase in the further course of the study.

#### Interim Sample and Flow Chart of DREAM_HAIR_

*N* = 603 participants, i.e., 317 families, were eligible for DREAM_HAIR_ as the anticipated delivery date was at least 4 weeks in the future (for flow chart of DREAM_HAIR_ see Supplementary Figure 1). Of those, *n* = 58 participants have been excluded at T1 due to drug intake, medical conditions, hair characteristics, or organizational reasons. These participants might be included again at future measurement points. Further *n* = 257 participants could not be enrolled because of lack of response or interest. For *n* = 136 participants T1 had not been due by the end of September 2018. As a result, the sample of DREAM_HAIR_ has consisted of *N* = 152 participants, i.e., 88 families, at this time. This is *n* = 87 expectant mothers, *n* = 64 male partners, and *n* = 1 female partner who returned hair samples at T1. As the T1 sampling of the child is due a few days after childbirth, *n* = 59 child samples (with one pair of twins) have been taken at T1 so far. In some cases, child hair was too short to be taken (*n* = 13). Further *n* = 17 child hair samples were not due yet.

T2 was due for *n* = 98 participants, i.e., 58 families from whom the majority (87.8%) returned T2 in time. Regarding child hair at T2, *n* = 47 samples out of *n* = 59 children (with one pair of twins) were returned. Further *n* = 6 child samples were not available because hair was too short to be taken and *n* = 6 samples were not returned in time. In addition, *n* = 9 participants, i.e., 5 families (with *n* = 5 children) joined the DREAM_HAIR_ study initially at T2 due to organizational reasons.

### Planned Data Analyses

The DREAM study provides quantitative data of four measurement points during the course of pregnancy to (at least) 2 years postpartum. Therefore, data will be analyzed both cross-sectionally and longitudinally with regard to the respective objectives of the study. More specifically, for the longitudinal analyses, multiple linear regression analyses, simple logistic regression analyses, and multinomial logistic regression analyses will be conducted to examine how prepartum individual and family factors predict outcomes after birth, such as health of family members and mothers' and their partners' participation in the labor market. As we are dealing with panel data, we will also perform fixed effects regression analyses in order to control for unobserved heterogeneity. Moreover, latent growth curve analyses in the framework of structural equation modeling (Bollen and Curran, 2006) and autoregressive cross-lagged analyses will be conducted when testing whether prepartum factors predict changes in social, work, and stress factors after birth. Full information maximum likelihood estimation or multiple imputation techniques will be used, as such techniques are considered to the most adequate approaches to handle missing data (Schafer and Graham, 2002).

We will analyze data on the individual level, while we will also have the opportunity to control for the partners' reports when both mother and partner participated as a couple. If both mother and her partner (and their baby, with respect to DREAM_HAIR_ and DREAM_MBU_) participate in the study, data sets can be combined using a family code.

Then, multi-level modeling as a beneficial statistical approach exceeding conventional methods will be applied, enabling us to analyze relations within and between dyads or triads while taking into account the shared variance in dyadic (couples) or triadic (couples and their child) structure of hierarchical data (Woltman et al., 2012; Davis et al., 2018). Multi-level modeling is also well-suited for examining changes over an extended period of time with several measurement points without being limited by missing data (Woltman et al., 2012).

#### Power Analyses

Power analyses for the basic DREAM study and DREAM_HAIR_ were conducted by means of Monte Carlo simulations using the R package “semsim,” version 0.5–13 (Beaujean, 2014). We conducted Monte Carlo simulation studies with 10,000 samples and examined the stability of our results by re-running the analyses with three different seeds. Power analyses were performed assuming a multivariate normal distribution of all variables. Moreover, we also estimated power with multivariate distributions deviating from normality. More specifically, in accordance with the methodological literature (Muthén and Asparouhov, 2002), we examined power for non-normal distributions with a skewness of 2.0 and a kurtosis of 3.5. Non-normal distributions were generated by using the multivariate Fleishman transformation (Vale and Maurelli, 1983). We also varied the proportion of missingness in the dependent variable (which is assumed to be measured at T4 where attrition is an important issue) from 0 to 70%.

First, we estimated power when the whole DREAM targeted sample of 2,000 couples, i.e., 4,000 individuals, are used in the analyses. With a sample size of *N* = 2,000, power analyses for multiple regression analyses with a continuous construct such as depressive symptoms as outcome variable (one of the main outcome variables in the study) and five continuous predictors showed a very high statistical power of 99% or above to detect significant effects given a small effect size of β = 0.20 between predictors and the outcome and a 5% (two-sided) type I error. Such high power was obtained even for non-normally distributed data and up to 70% proportion of missing data in the outcome variable. Therefore, the sample size at T4 will be large enough to obtain very high statistical power even with a dropout rate of 70% over the four assessment points.

As the number of same-sex couples in our study is very small, our statistical power will most likely not be sufficient to perform subgroup analyses. Therefore, we would consider these family constellations as case studies, which can be used to generate hypotheses for further studies with a targeted focus on families with same-sex parents.

Regarding the sub-study DREAM_HAIR_, approximately 20% of participants of the basic DREAM study participate in DREAM_HAIR_; hence we estimate *N* = 360 couples, i.e., 720 individuals, will participate at T1. For *N* = 360, even with a dropout rate of 40% at T4, Monto Carlo simulations estimated a power of 93% (multivariate normal distribution) and 80% (non-normally distributed data) to detect small effect sizes of β = 0.20 between predictors and the outcome in multiple regression analyses with five predictors and a 5% (two-sided) type I error. Moreover, a somewhat larger effect size of β = 0.28 will be detected with a power of 94% (multivariate normal distribution) and a power of 80% (non-normally distributed data) in the unlikely case that attrition will be as high as 70%.

With respect to the sub-study DREAM_MBU_, the clinical sample will be much smaller (*N* = 70 mothers with their babies and, if available, partners). We expect a higher compliance of the clinical branch (compared to the community branch), as women are personally bound to our team. Specifically, this sample size allows to detect medium effects at an α-level of 0.05 and a statistical power of 0.80 (repeated measures ANOVA, four measurement points), assuming a moderate correlation (*r* = 0.50) among repeated measures as conducted using G^*^Power 3.1 (Faul et al., 2007, 2009). As we will be able to recruit participants for the clinical sample for a longer period than for the community sample, our sample size then will be sufficient to allow subgroup analyses as a function of responder status or pre-treatment HCC.

Regarding DREAM_TALK_, we are targeting *N* = 40 couples. In qualitative research, the sample size can generally be determined by saturation. While the sample size should be large enough to sufficiently describe the phenomenon of interest (Glaser and Strauss, 1967; Malterud et al., 2016), saturation is reached when no further information is gained by interviewing further participants (Kvale, 1996). Different authors have suggested sample sizes between 20 and 50 (Morse, 1994; Creswell and Poth, 2013). Therefore, we are confident that our subsample size will be large enough to reach saturation.

Altogether, the targeted sample size of the basic DREAM study and the subsample sizes of the sub-studies will be large enough to answer the main research questions.

## First Results

In this chapter, preliminary results regarding the sample of the basic DREAM study are presented.

### Sociodemographic Characteristics

Tables 3, 4 show preliminary sociodemographic characteristics of the basic DREAM study sample during pregnancy (T1). The majority of expectant women (96.0%), male partners (95.6%), and all female partners reported German to be the mother tongue. On average, at T1 expectant mothers were in gestational week 29.9 (*SD* = 6.1; *Range* = 10–41) and most had a singleton pregnancy (98.0%) while *n* = 11 women were pregnant with twins and *n* = 2 women with multiples. For the majority of them, it was the first child (expectant mothers: 78.3%; male partners: 76.0%; female partners: 77.8%).

**Table 3:** Sociodemographic characteristics of expectant mothers and their partners during pregnancy (T1).

	**Expectant mothers** **(*n* = 805)**	**Male partners to the mother** **(*n* = 596)**	**Female partners to the mother** **(*n* = 9)**
**Age**	30.1 ± 4.0 (15-42)	32.4 ± 5.0 (22-56)	35.4 ± 6.5 (28-47)
**Marital status**			
Married/registered same sex partnership	336 (41.7)	259 (43.4)	8 (88.9)
Unmarried	441 (54.8)	311 (52.2)	1 (11.1)
Divorced	25 (3.1)	22 (3.7)	0 (0.0)
Widowed	0 (0.0)	0 (0.0)	0 (0.0)
Unknown	2 (0.3)	0 (0.0)	0 (0.0)
Missing data	1 (0.1)	4 (0.7)	0 (0.0)
**Partnership**			
Yes	791 (98.3)	588 (98.7)	9 (100.0)
**Living together**			
Yes, permanently	745 (94.2)	555 (94.4)	9 (100.0)
Yes, not permanently	35 (4.4)	26 (4.4)	0 (0.0)
No	7 (0.9)	5 (0.9)	0 (0.0)
Missing data	4 (0.5)	2 (0.3)	0 (0.0)
No	9 (1.1)	2 (0.3)	0 (0.0)
Missing data	5 (0.6)	6 (1.0)	0 (0.0)
**Number of children**			
0	630 (78.3)	453 (76.0)	7 (77.8)
1	143 (17.8)	101 (17.0)	1 (11.1)
2	20 (2.5)	24 (4.0)	0 (0.0)
3	4 (0.5)	4 (0.7)	0 (0.0)
4	2 (0.2)	0 (0.0)	0 (0.0)
Missing data	6 (0.7)	14 (2.3)	1 (11.1)
**Education**			
No degree	0 (0.0)	2 (0.3)	0 (0.0)
Lower secondary education level 2	7 (0.9)	16 (2.7)	0 (0.0)
Secondary school certificate	164 (20.4)	157 (26.3)	5 (55.6)
Advanced technical college entrance qualification	66 (8.2)	51 (8.6)	2 (22.2)
A-level through second chance education	17 (2.1)	16 (2.7)	0 (0.0)
Subject-related or higher education entrance qualification (A-level)	548 (68.1)	341 (57.2)	2 (22.2)
Still in school	2 (0.2)	0 (0.0)	0 (0.0)
Unknown	1 (0.1)	2 (0.3)	0 (0.0)
Missing data	0 (0.0)	11 (1.9)	0 (0.0)
**Professional qualification**			
No qualification	4 (0.5)	4 (0.7)	0 (0.0)
Occupational apprenticeship	285 (35.4)	198 (33.2)	6 (66.7)
Master of crafts	30 (3.7)	47 (7.9)	1 (11.1)
University	434 (53.9)	277 (46.4)	2 (22.2)
Doctoral degree	34 (4.2)	32 (5.4)	0 (0.0)
Still in qualification	15 (1.9)	20 (3.4)	0 (0.0)
Missing data	3 (0.4)	18 (3.0)	0 (0.0)
**Working hours per week**	37.5 ± 8.9 (2-68)	40.9 ± 7.7 (1-70)	40.7 ± 3.7 (35-45)
**Net earnings on average**			
Up to 450 €	25 (3.1)	14 (2.3)	0 (0.0)
451 € to 850 €	25 (3.1)	11 (1.9)	0 (0.0)
851 € to 1,500 €	224 (27.8)	106 (17.8)	4 (44.4)
1,501 € to 2,500 €	417 (51.8)	307 (51.5)	5 (55.6)
More than 2,500 €	73 (9.1)	125 (21.0)	0 (0.0)
Not working or missing data	41 (5.1)	33 (5.5)	0 (0.0)
**Job burden**			
Not burdened	73 (9.1)	54 (9.1)	0 (0.0)
A little bit burdened	254 (31.5)	163 (27.3)	2 (22.2)
Moderately burdened	269 (33.4)	254 (42.6)	3 (33.4)
Heavily burdened	100 (12.4)	79 (13.3)	2 (22.2)
Very heavily burdened	7 (0.9)	11 (1.8)	1 (11.1)
Not working or missing data	102 (12.7)	35 (5.9)	1 (11.1)
**Job satisfaction**			
Very satisfied	103 (12.8)	94 (15.8)	1 (11.1)
Quite satisfied	323 (40.1)	282 (47.3)	4 (44.5)
Neither unsatisfied nor satisfied	132 (16.4)	111 (18.6)	2 (22.2)
Quite unsatisfied	94 (11.7)	57 (9.6)	1 (11.1)
Very unsatisfied	48 (6.0)	18 (3.0)	1 (11.1)
Not working or missing data	105 (13.0)	34 (5.7)	0 (0.0)
**Intention to take parental leave**			
Yes	757 (94.0)	477 (80.0)	7 (77.8)
No	5 (0.6)	61 (10.3)	2 (22.2)
Unknown	2 (0.3)	27 (4.5)	0 (0.0)
Missing data	41 (5.1)	31 (5.2)	0 (0.0)
**Duration of parental leave as intended**	14.4 ± 5.0 (3-36)	3.3 ± 3.0 (1-24)	4.4 ± 3.7 (2-12)

*n (%) or M **±** SD (Range)*.

**Table 4:** Former (1 year ago) and current employment during pregnancy (T1).

	**Expectant mothers (*n* = 805)**	**Male partners to the mother (*n* = 596)**	**Female partners to the mother (*n* = 9)**
	***n (%)***	***n (%)***	***n (%)***
**Former employment (1 year ago)**			
No employment	9 (1.1)	5 (0.8)	0 (0.0)
Not regularly employed	8 (1.0)	6 (1.0)	0 (0.0)
Marginal employment	39 (4.8)	23 (3.9)	0 (0.0)
Part-time	185 (23.0)	41 (6.9)	0 (0.0)
Full-time	498 (61.9)	484 (81.2)	8 (88.9)
Occupational retraining	0 (0.0)	1 (0.2)	0 (0.0)
Volunteer	2 (0.2)	0 (0.0)	0 (0.0)
Housewife/houseman	3 (0.4)	2 (0.3)	0 (0.0)
Parental leave	20 (2.5)	1 (0.2)	0 (0.0)
Still in school	3 (0.4)	1 (0.2)	0 (0.0)
Still in apprenticeship	17 (2.1)	6 (1.0)	0 (0.0)
Still in university	86 (10.7)	59 (9.9)	0 (0.0)
Employment ban	8 (1.0)	0 (0.0)	0 (0.0)
Occupational disability	3 (0.4)	0 (0.0)	0 (0.0)
Other	18 (2.2)	9 (1.5)	0 (0.0)
Missing data	11 (1.4)	7 (1.2)	1 (0.0)
**Current employment**			
No employment	17 (2.1)	5 (0.8)	0 (0.0)
Not regularly employed	2 (0.2)	3 (0.5)	0 (0.0)
Marginal employment	21 (2.6)	17 (2.9)	0 (0.0)
Part-time	135 (16.8)	44 (7.4)	1 (11.1)
Full-time	359 (44.6)	492 (82.6)	8 (88.9)
Occupational retraining	0 (0.0)	2 (0.3)	0 (0.0)
Volunteer	1 (0.1)	0 (0.0)	0 (0.0)
Housewife/houseman	9 (1.1)	1 (0.2)	0 (0.0)
Parental leave	103 (12.8)	1 (0.2)	0 (0.0)
Still in school	3 (0.4)	0 (0.0)	0 (0.0)
Still in apprenticeship	6 (0.7)	6 (1.0)	0 (0.0)
Still in university	62 (7.7)	47 (7.9)	0 (0.0)
Employment ban	239 (29.7)	0 (0.0)	0 (0.0)
Occupational disability	3 (0.4)	0 (0.0)	0 (0.0)
Other	24 (3.0)	8 (1.3)	0 (0.0)
Missing data	1 (0.1)	4 (0.7)	0 (0.0)

*Multiple answers were possible*.

Mean age of expectant mothers was 30.1 years (*SD* = 4.0; *Range* = 15–42), 32.4 years of male partners (*SD* = 5.0; *Range* = 22–56), and 35.4 years of female partners (*SD* = 6.5; *Range* = 28–47).

Compared to the overall German population (Statistisches Bundesamt, 2018a,b) and the population of Dresden (Statistisches Landesamt Sachsen, 2018), participants are characterized by a rather high educational and professional level and full-time work status.

Regarding employment 1 year ago, i.e., prior to pregnancy, first analyses showed that a greater ratio of male partners (81.2%; *n* = 484) than expectant mothers (61.9%; *n* = 498) had a full-time position (χ^2^ = 62.153; *df* = 1; *p* < 0.01). Accordingly, a part-time position was hold by a greater ratio of expectant mothers (23.0%; *n* = 185) than male partners (6.9%; *n* = 41; χ^2^ = 66.032; *df* = 1; *p* < 0.01). These gender differences could be found irrespective of whether expecting the first child or already having children, i.e., the ratio holding a full-time position was lower in first-time mothers (72.1%; *n* = 446) compared to first-time fathers (80.4%; *n* = 360; χ^2^ = 9.702; *df* = 1; *p* < 0.01) as well as in mothers (28.4%; *n* = 48) compared to fathers already having children (87.4%; *n* = 111; χ^2^ = 101.521; *df* = 1; *p* < 0.01). Likewise, the ratio working part-time was higher both in first-time mothers and mothers already having children compared to the respective men.

In fact, as shown in Table 5, these gender differences regarding employment 1 year ago, i.e., prior to pregnancy, were more pronounced in parents who already had children compared to first-time parents. Hence, among expectant mothers, first-time mothers (72.1%; *n* = 446) were working full-time more often than mothers already having children (28.4%; *n* = 48; χ^2^ = 108.140; *df* = 1; *p* < 0.01). Regarding part-time work, it was vice-versa, i.e., a higher ratio of mothers already having children (47.9%; *n* = 81) compared to first-time mothers (16.5%; *n* = 102) was working part-time (χ^2^ = 73.648; *df* = 1; *p* < 0.01). In male partners, no such a difference between first-time fathers and fathers already having children could be found regarding a full-time (80.4%; *n* = 360 vs. 87.4%; *n* = 111; χ^2^ = 3.314; *df* = 1; *p* = 0.07) or part-time position (7.1%; *n* = 32 vs. 7.1%; *n* = 9; (χ^2^ = 0.000; *df* = 1; *p* = 0.98).

**Table 5:** Comparisons between sexes and first-time vs. parents already having children regarding full-time and part-time work status 1 year ago, i.e., prior to pregnancy (T1).

	**Compared groups**	**Work status**	***n (%)***	**χ*^**2**^***	***df***	***p***
Comparison between sexes	EM_0_ vs. P_0_	Full-time	EM_0_: 446 (72.1%) P_0_: 360 (80.4%)	9.702	1	0.00
		Part-time	EM_0_: 102 (16.5%) P_0_: 32 (7.1%)	20.626	1	0.00
	EM_1+_ vs. P_1+_	Full-time	EM_1+_: 48 (28.4%) P_1+_: 111 (87.4%)	101.521	1	0.00
		Part-time	EM_1+_: 81 (47.9%) P_1+_: 9 (7.1%)	57.160	1	0.00
Comparison between first-time parents and parents already having children	EM_0_ vs. EM_1+_	Full-time	EM_0_: 446 (72.1%) EM_1+_: 48 (28.4%)	108.140	1	0.00
		Part-time	EM_0_: 102 (16.5%) EM_1+_: 81 (47.9%)	73.648	1	0.00
	P_0_ vs. P_1+_	Full-time	P_0_: 360 (80.4%) P_1+_: 111 (87.4%)	3.314	1	0.07
		Part-time	P_0_: 32 (7.1%) P_1+_: 9 (7.1%)	0.000	1	0.98

*At T1 (during pregnancy), participants were asked whether they have been working full-time or part-time 1 year ago, i.e., prior to pregnancy. EM0: first-time expectant mothers; EM1+: expectant mothers already having children; P0: first-time fathers (male partners); P1+: fathers (male partners) already having children*.

Regarding current employment, i.e., during pregnancy, nearly one third of expectant mothers was in employment ban because of a health risk for mother or child (29.7%; *n* = 239). Further, the number of expectant mothers currently holding a full-time (44.6%; *n* = 359; **χ**^2^ = 101.327; *df* = 1; *p* < 0.01) and part-time position (16.8%; *n* = 135; **χ**^2^ = 23.539; *df* = 1; *p* < 0.01) was lower than 1 year ago (i.e., prior to pregnancy). In male partners, no such decrease regarding a full-time (**χ**^2^ = 1.333; *df* = 1; *p* = 0.25) and part-time position (exact *p* = 0.63) within the last year was found.

During pregnancy, the majority in all groups reported that they intended to take parental leave. Expectant mothers and male partners differed in the intention to take parental leave (94.0 vs. 80.0%; **χ**^2^ = 105.16, *df* = 2; *p* < 0.01). Further, expectant mothers intended to take parental leave for a longer period of time (*M* = 14.4 months; *SD* = 5.0; *Range* = 3–36) than male partners (*M* = 3.3 months; *SD* = 3.0; *Range* = 1–24; *t*_(1209.48)_ = 48.57, *p* < 0.01, *BCa 95%-CI* [10.63; 11.56]).

### Birth-Related Characteristics

By the end of September 2018, birth-related characteristics at T2 (Table 6) were available for *n* = 465 mothers who had given birth to their child during the period from July 2017 to July 2018 with preterm delivery in *n* = 13 cases (2.8%) which is lower than in the overall German population (in 2013: 8.7%, Statistisches Bundesamt; born in hospitals in 2017: 8.6 %, IQTIG–Institut für Qualitätssicherung und Transparenz im Gesundheitswesen, 2018). *N* = 465 mothers gave birth to *n* = 472 children with a nearly balanced gender ratio (51.5%; *n* = 243 female) which is representative for the overall German population (48.7% female) and Saxony (48.9% female) in 2017 (Statistisches Bundesamt, 2018d). Most of the children were delivered vaginally (80.6%; *n* = 375). The ratio of cesarean sections (18.5%; *n* = 86) was as low as it is typical for the area of Dresden (in 2013: 19.5%; Statistisches Bundesamt, 2015), i.e., lower than in Saxony (24.0%) and in the overall German population (30.5%) in 2017 (Statistisches Bundesamt, 2018c). The majority of partners attended birth (97.3%; *n* = 319 male partners and 100%; *n* = 3 female partners).

**Table 6:** Birth-related characteristics as reported by the mothers 8 weeks after anticipated birth date (T2).

	**Mothers (*n* = 465)**
	***n (%)***
**Number of delivered children**
One	459 (98.7)
Twins	5 (1.1)
Multiples	1 (0.2)
**Preterm delivery**
Yes	13 (2.8)
No	445 (95.7)
Missing data	7 (1.5)
**Mode of delivery**
Spontaneous vaginal birth	257 (55.3)
Vaginal birth induced by drugs	81 (17.4)
Vaginal operative birth (with forceps or vacuum extraction)	37 (7.9)
Planned cesarean section due to personal reasons	2 (0.4)
Planned cesarean section due to medical reasons	31 (6.7)
Unplanned cesarean section	53 (11.4)
Missing data	4 (0.9)
**Sex of child (*****n*** **=** **472)**
Female	243 (51.5)
Male	221 (46.8)
Missing data	8 (1.7)

### Dropout Analyses

Dropout analyses (tables on request) considering sociodemographic characteristics of completers vs. non-completers (whose T2 data were due by the end of September 2018) were conducted separately for expectant mothers (*n* = 465 vs. *n* = 96) and male partners (*n* = 328 vs. *n* = 90), but not for female partners due to the small sample sizes (*n* = 3 vs. *n* = 3). Completers and non-completers at T2 did not differ in sociodemographic characteristics with some exceptions.

Regarding expectant mothers, completers were reporting German as their mother tongue (96.8 vs. 91.7%; Fisher's exact test, *p* < 0.05) and intended to take parental leave (99.3 vs. 95.5%; Fisher's exact test, *p* < 0.01) more often than non-completers. While there were no differences regarding current employment, completers differed from non-completers regarding employment 1 year ago. Formerly, completers were working in a full-time position more often than non-completers (65.4 vs. 53.7%; **χ**^2^ = 4.674, *df* = 1, *p* < 0.05), while the ratio of not being regularly employed (0.2 vs. 4.2%; Fisher's exact test, *p* < 0.01) and being a housewife (0.0 vs. 2.1%; Fisher's exact test, *p* < 0.05) was lower in completers than in non-completers.

Regarding male partners, completers lived permanently together with the expectant mother more often (96.3 vs. 88.8%; Fisher's exact test, *p* < 0.05), had a higher education (e.g., 61.6 vs. 51.1% having a subject-related or higher education entrance qualification (A-level); *U* = 12113.0; *z* = −2.140; *p* < 0.05), and a higher professional qualification (e.g., 57.0 vs. 40.0% having a university or doctoral degree; *U* = 11458.0; *z* = −2.424; *p* < 0.05) than non-completers.

## Discussion

Examining expectant mothers and their partners from pregnancy to at least 2 years postpartum, the DREAM study is one of the first prospective cohort studies investigating the changes of parental work participation and conditions, role distributions, and stress factors and its impact on perinatal outcomes and long-term mental and somatic health of the entire family while taking into account underlying biological processes regarding long-term activity of the HPA axis.

Altogether, major strengths of the DREAM study are (1) the prospective design stretching from one measurement point prepartum to at least three additional measurement points within the first two years postpartum (prolongation into middle childhood planned) which allows both longitudinal and cross-sectional analyses, (2) a unique multi-method approach combining quantitative data (using predominantly well-established scales) with long-term endocrinological data (DREAM_HAIR_) and qualitative interview data (DREAM_TALK_), (3) a large, representative community sample (interim sample of *N* = 1,410 participants by the end of September 2018, targeting *N* = 4,000 participants) of both mothers and male or female partners allowing the use of advanced statistical approaches such as multi-level modeling, (4) comparison to a clinical sample of mothers with postpartum mental disorders, their children, and (if available) their partners during the course from admission to discharge of a MBU tracking intervention effects and post-treatment, (5) a cooperation within Germany and abroad with matching instruments and assessment waves to facilitate internal and cross-cultural comparisons of findings.

Regarding the interim sample, first results of analyses conducted for this paper showed that our interim sample mainly consists of expectant mothers and their partners who expect their first child. This is because some of the recruitment ways like the clinics' information evenings or birth preparation courses are visited mainly by families expecting their first child. Nevertheless, it is exactly those parents who are of main interest, as the present study aims primarily at investigating the transition and change from being a couple to becoming a family. Further, there are slightly more expectant mothers than partners participating and the majority of participants has a rather high educational and professional level. This is in accordance with previous findings, i.e., people are more likely to participate in epidemiologic studies if they are female, well-educated, and/or have a higher socioeconomic status (e.g., Søgaard et al., 2004; Galea and Tracy, 2007; Gustavson et al., 2012). The self-selection bias might be even bigger in family studies. First, fathers might join or complete those studies more seldom than mothers. Second, particularly those fathers who do participate might be more involved in family matters or might have more progressive gender role values than nonparticipating fathers as discussed in Costigan and Cox (2001). Also, it is difficult to reach and recruit becoming fathers who are not in a relationship with the becoming mother or who are not accompanying their partners at the information evening or birth preparation courses. Moreover, it is conceivable that families interested in the compatibility of work and family may have contributed to the high ratio of expectant mothers and partners holding a full-time position. When speaking about the representativeness of the sample and generalizability of findings, the possibility of a healthy worker-effect has to be considered, i.e., in research on working populations, healthy and little burdened individuals are often overrepresented due to their greater likelihood of participating in workforce (Li and Sung, 1999; Shah, 2009).

Further, we showed that a higher ratio of first-time mothers has been working full-time prior to pregnancy compared to mothers already having a child/children. No such difference was found in male partners. This is in accordance with previous findings that German women but not men reduce their amount of working time after giving birth to a child (Bundesministerium für Familie Senioren Frauen und Jugend, 2017a). More importantly, this is in line with our postulation of an emerging gender gap interfering with gender equality as it has consequences for long-term labor market inclusion/exclusion (Barker and Pawlak, 2011; Miani and Hoorens, 2014). Altogether, this sample seems to be appropriate for examining our research questions as a pronounced number of working participants is needed to profoundly investigate the changes of parental work participation, role distributions, and stress factors and its long-term implications for family health.

Regarding birth-related characteristics, first analyses showed that the ratio of mothers who delivered by cesarean section is as low as typical for the region (Statistisches Bundesamt, 2015) while the rate of preterm deliveries of the interim DREAM sample is lower compared to the overall German population (Statistisches Bundesamt, 2015; IQTIG–Institut für Qualitätssicherung und Transparenz im Gesundheitswesen, 2018). A possible reason may be that participating mothers who had a preterm delivery were more likely to drop out because child care is more extensive in the early postpartum, i.e., an already sensitive period.

Dropout analyses found only a few differences between completers and non-completers at T2 indicating that (1) non-completing male partners had a lower educational level as well as professional qualification and lived more seldom permanently together compared to completing male partners and (2) non-completing mothers were speaking German as their mother tongue, working full-time, and planning to take parental leave less often than completing mothers. However, the DREAM study is characterized by a rather low dropout rate which is supported by our retention strategy using reminders and incentives (Booker et al., 2011). In future, we will still increase personal contact via telephone to participants who do not send back the follow-up questionnaires in time to reduce the attrition even more. This will be an additional strength in the further course of the study as we prospectively would like to add measurement points reaching into middle childhood. Hence, it will be possible to examine long-term effects on family health as well as on a potential gender gap regarding labor market inclusion/exclusion (Schober and Zoch, 2015).

To sum up, the DREAM study will contribute to a better understanding of the complex relationships between parental work participation, role distribution, and stress factors and its implications for perinatal outcome and long-term mental and somatic health of mothers, their partners in parenting, and children. With former studies neglecting confounding and moderating factors, this innovative study with its sub-studies allows a comprehensive picture of the family as a whole. To our knowledge, it is the first study combining work-related health implications with the assessment of long-term activity of the HPA axis as measured by HCC in each individual and parent-child dyad. In particular, examining the cumulated steroid hormones of both parents and their child in HCC data, the current findings will be valuable for understanding the transgenerational transmissions of long-term alterations of the HPA axis and the interplay as a couple. The comparison to a clinical sample of mothers, their children, and (if available) their partners who will be assessed during the course of a state-of-the-art treatment in a MBU will help to better understand the potential underlying mechanisms of these processes and intervention benefits. The clinical sub-study has further the potential to add evidence regarding the role of parent-child interaction for child health, particularly its temperament and regulation disorders. Moreover, it is unique to this research area to investigate both mother-child and father-child dyads. Finally, results will not only be of scientific interest but also of socio-political relevance as they will generate important findings warranted by this inter-disciplinary research field and thus may contribute moving these issues higher on the political agenda.

## Ethics Statement

This study has been carried out in accordance with the recommondations of the Ethics Committee of the Faculty of Medicine of the Technische Universität Dresden with written consent from all subjects. All subjects gave written informed consent in accordance with the Declaration of Helsinki. This protocol was approved by the DREAM steering group and by the Ethics Committee of the Faculty of Medicine of the Technische Universität Dresden (No: EK 278062015).

## Author Contributions

SG-N has acquired the funding and been responsible for conception and design of the basic DREAM study with its sub-studies as well as the coordination and supervision of the (ongoing) data collection. JJ-H, SS-S, SG-N, and AF have been responsible for the conception and design of the sub-study DREAM_HAIR_. MK, VK, CA, CS, and AF supported the conduction of the study, especially through data collection. PW provided access to potential participants. KW provided resources for the acquisition of data in the DREAM study. CK advised the sub-study DREAM_HAIR_ and provided support for hair cortisol analyses. MK and VK prepared the data for statistical analysis. VK performed the statistical analysis except from Monte Carlo simulations. TvS performed Monte Carlo simulations. VK wrote the first draft of the manuscript. All authors contributed to manuscript revision, read, and approved the submitted version.

### Conflict of Interest Statement

The authors declare that the research was conducted in the absence of any commercial or financial relationships that could be construed as a potential conflict of interest.
