# Supplementary material for: The Impact of Parental Role Distributions, Work Participation, and Stress Factors on Family Health-Related Outcomes: Study Protocol of the Prospective Multi-Method Cohort “Dresden Study on Parenting, Work, and Mental Health” (DREAM)
Source: Front Psychol. 2019 Jun 12;10:1273. doi: 10.3389/fpsyg.2019.01273 (PMC6584823; doi:10.3389/fpsyg.2019.01273)
Supplement: Supplementary file 1 [file Table_1.DOCX]

Supplementary Material

**The impact of parental role distributions, work participation, and stress factors on family health-related outcomes: Study protocol of the prospective multi-method cohort “Dresden Study on Parenting, Work, and Mental Health” (DREAM)**

Victoria Kress^1^, Susann Steudte-Schmiedgen^1,2^, Marie Kopp^1^, Anke Förster^1^, Caroline Altus^1^, Caroline Schier^1^, Pauline Wimberger^3^, Clemens Kirschbaum^2^, Kerstin Weidner^1^, Juliane Junge-Hoffmeister^1^, Susan Garthus-Niegel^1,4*^

^1^Department of Psychotherapy and Psychosomatic Medicine, Faculty of Medicine of the Technische Universität Dresden, Dresden, Germany

^2^Department of Biological Psychology, Faculty of Psychology of the Technische Universität Dresden, Dresden, Germany

^3^Department of Gynecology and Obstetrics, Faculty of Medicine of the Technische Universität Dresden, Dresden, Germany

^4^Department of Child Health, Norwegian Institute of Public Health, Oslo, Norway

**^*^ Correspondence:**Susan Garthus-Niegel
susan.garthus-niegel@uniklinikum-dresden.de

**Supplementary Figure 1.** Flow chart of the long-term endocrinological sub-study DREAM_HAIR_. Notes: T1 parents during pregnancy and children up to 2 weeks postpartum, T2 8 weeks after anticipated birth date. Data from end of September 2018 (data collection is not finished yet, recruitment ongoing). Further future assessment waves (not due yet): T3 14 months and T4 2 years after actual birth date. ^a^Participants might be included again at future measurement points. ^b^Child samples include samples of one pair of twins.
