# Supplementary figures and images for: The Impact of Parental Role Distributions, Work Participation, and Stress Factors on Family Health-Related Outcomes: Study Protocol of the Prospective Multi-Method Cohort “Dresden Study on Parenting, Work, and Mental Health” (DREAM)
Source: Front Psychol. 2019 Jun 12;10:1273. doi: 10.3389/fpsyg.2019.01273 (PMC6584823; doi:10.3389/fpsyg.2019.01273)

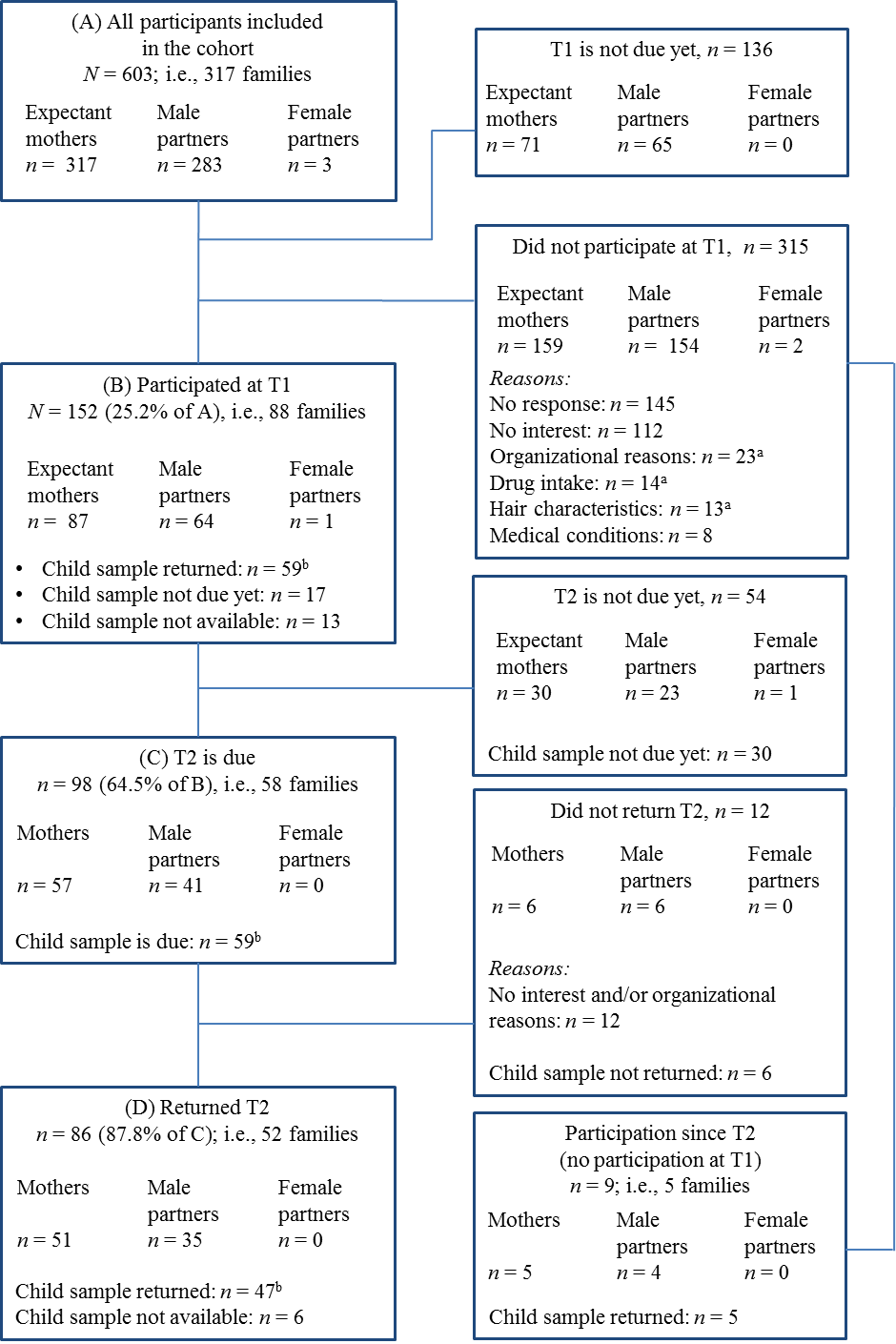

Supplement: Supplementary file 2 [file Image_1.TIF]
